# Editorial: Myelin in white mater function and related diseases

**DOI:** 10.3389/fnmol.2023.1284273

**Published:** 2023-09-12

**Authors:** Zhen Tian, An Cheng

**Affiliations:** ^1^College of Pharmaceutical Sciences, Southwest University, Chongqing, China; ^2^Department of Ophthalmology, School of Medicine, University of California, San Francisco, San Francisco, CA, United States

**Keywords:** myelin, oligodendrocyte, traumatic brain injury, spinal cord injury, Tourette syndrome, inflammatory pain, major depressive disorder

Oligodendrocytes are the myelinating cells of the central nervous system (CNS) that constitute about 5–10% of the total glial population (Yeung et al., 2019). Myelin is a lipid-rich substance generated by oligodendrocytes (Masson and Nait-Oumesmar, 2023). The extension of the plasma membrane of oligodendrocytes shapes myelin sheath, which wraps around nerve axons in a concentric manner (Kuhn et al., 2019). The myelin sheath not only facilitates the rapid and efficient conduction of the electrical impulses along the axons, but also offers metabolic support to the axons it ensheaths and maintains axonal integrity (Nave, 2010). Oligodendrocytes are indispensable to myelin formation in the developing CNS and crucial for myelin regeneration following injury (Franklin and Ffrench-Constant, 2017). The disruption in myelination during development, adaptive myelination in adulthood or remyelination after injury all can lead to neurological dysfunction. For example, the impairment in oligodendrocyte function and in myelination can potentiate the emergence of neurological disorders such as schizophrenia, multiple sclerosis and Alzheimer's disease (Barateiro et al., 2016). The loss of oligodendrocytes and demyelination of spared axons contribute to the motor, sensory and autonomic impairments of spinal cord injury (Pukos et al., 2019).

The main aim of this Research Topic was to invigorate the research in studying the role of myelin and oligodendrocytes in health and diseases. This Research Topic brings together five original research articles investigating traumatic brain injury, Tourette syndrome, spinal cord injury, inflammatory pain and major depressive disorder as well as a bibliometric analysis of gene expression in spinal cord injury. All these disorders are at least partially attributed to the damage in oligodendrocyte function and myelination.

Iron overload contributes to the neuronal dysfunction following traumatic brain injury (TBI). Cheng et al. investigated the spatial-temporal iron metabolism and deposition in neuronal cells after TBI. They found that iron overload and ferroptotic pathology were observed in the ipsilateral cortical neurons at early phase of TBI (1–3 days post-injury). However, iron accumulation was absent in astrocytes. Interestingly, iron deposition was only observed at 7–14 days post-injury in oligodendrocytes, which was in accordance with the corresponding interval of cellular repair. Their results suggest that iron deposition and metabolism is different in neurons and glial cells after TBI, namely it exhibited cell type-specific spatial-temporal changes.

Wang et al. performed a bibliometric analysis of gene expression in spinal cord injury (SCI). They found that the number of annual publications related with SCI increased in general. The molecular and pathological mechanisms as well as novel therapies for SCI were the main theme of these articles. The core of each hotspot, such as neuropathic pain, axonal regeneration, transplantation, and functional recovery, may represent the promising directions and are probably the future trend of this field. Their study may offer references for choosing topics in future researches, especially the leading-edge development in the field of SCI.

Tourette syndrome (TS) is a neurodevelopmental disorder with recurrent motor and vocal tics as the core symptoms. The structural and functional abnormalities in the cortico-basal ganglia circuitry were also observed in the TS. The inactivating mutation of *histidine decarboxylase gene* (*Hdc*) has been identified as a genetic cause of TS. Jindachomthong et al. examined the white matter abnormalities in the *Hdc* knockout mouse. They demonstrated that the genes associated with oligodendrocytes and myelin production (e.g., Myelin Basic Protein, MBP; Myelin Associated Oligodendrocyte Basic Protein, MOBP; Oligodendrocyte transcription factor 2, Olig2; and 2′, 3′-Cyclic nucleotide 3′-phosphodiesterase, CNP) are increased in the dorsal striatum of *Hdc* knockout mice. Their results indicate that the *Hdc*-KO mouse may represent a good animal model to examine the developmental abnormalities of TS.

Traditional Chinese medicine may provide an option for the treatment of a variety of central nervous system disorders. Fu et al. combined the network pharmacology and experimental methods to examine the therapeutic activity and underlying mechanism of Epimedium (EPI), a common Chinese herb, for spinal cord injury (SCI). They showed that EPI improved the behavioral outcomes of SCI rats significantly. The mechanism study revealed that EPI reduced the level of malondialdehyde (MDA) and increased the level of superoxide dismutase (SOD), and glutathione (GSH), while this phenomenon could be blocked by PI3K inhibitor, implying that EPI could inhibit the oxidative stress via activation of the PI3K/AKT signaling pathway in SCI rats. Their results indicated that EPI may be a potential candidate for the treatment of SCI.

Chronic inflammation may induce spinal central sensitization and then result in pathological pain. Ducza et al. investigated the role of ionotropic P2X purino receptor 4 (P2X4) in peripheral inflammatory pain in rat spinal dorsal horn. They demonstrated that the mRNA, protein as well as the immunoreactivity of P2X4 was all enhanced significantly within the spinal dorsal horn of CFA-induced inflammatory pain rats. The robust protein increase was mainly detected on primary afferent axon terminals and GFAP-labeled astrocyte membrane compartments, but not on postsynaptic dendrites. In addition, abundant P2X4 receptors were also observed in the peptidergic and non-peptidergic nociceptive subsets of ganglia cells in lumber DRG. Their results suggest that neuronal and glial P2X4 receptor contributes to the establishment of inflammatory pain.

Abnormalities in myelin are one of the important contributors of major depressive disorder (MDD). Using new quantitative myelin-related maps of high-resolution 7 Tesla (7 T) magnetic resonance imaging, Shim et al. assessed the difference in myelin concentration in the white matter and subcortical areas between healthy controls and patients with MDD. They found that the average smoothed quantitative ratio (sq-ratio) and sq-ratio myelin-related values in the fornix of white matter and thalami were higher in the healthy controls than in the MDD patients. Their results indicate that the myelin concentration in white matter and subcortical areas of MDD patients is reduced.

## Author contributions

ZT: Conceptualization, Funding acquisition, Validation, Writing—original draft. AC: Conceptualization, Validation, Writing—review and editing.
